# 109 The Impact of Distance to Treatment Center on Long-term Outcomes of Burn Patients

**DOI:** 10.1093/jbcr/irac012.112

**Published:** 2022-03-23

**Authors:** Kevin E Galicia, John Kubasiak, Anupama Mehta, Robert Riviello, Stephanie Nitzschke, Kara McMullen, Nicole S Gibran, Barclay T Stewart, Steven E Wolf, Colleen M Ryan, Jeffrey C Schneider

**Affiliations:** Loyola University Medical Center, Maywood, Illinois; Loyola University Medical Center, Maywood, Illinois; Brigham and Women's Hospital, Quincy, Massachusetts; Brigham and Women's Hospital, Brookline, Massachusetts; Brigham and Women's Hospital, Boston, Massachusetts; University of Washington, Portland, Oregon; University of Washington, Wellfleet, Massachusetts; University of Washington, Seattle, Washington; University of Texas Medical Branch at Galveston, Galveston, Texas; Harvard Medical School, Boston, Massachusetts; , Massachusetts

## Abstract

**Introduction:**

Geospatial access to American Burn Association (ABA)-verified burn centers or self-designated burn care facilities varies across the country. It is often necessary to transport patients hundreds of miles to provide definitive burn care and rehabilitation services. This study evaluates the impact of distance to treatment center on long-term outcomes of burn patients.

**Methods:**

Data from the National Institute on Disability, Independent Living, and Rehabilitation Research Burn Model System (BMS) National Database, collected from 2015 to 2019, were analyzed to investigate the impact of distance to BMS center on long-term, patient-reported outcomes. Distance was calculated as driving distance between home zip code centroid and BMS center. Demographic and clinical data were compared between groups by distance from BMS center (< 20, 20-49.9, >50 miles). The following patient-reported outcome measures, collected 12 months after injury, were examined: Veterans Rand 12 Physical Component Summary Score (VR-12 PCS), Veterans Rand 12 Mental Component Summary Score (VR-12 MCS), Satisfaction with Life (SWL), employment status, and days to return to work. Mixed regression model analyses were used to examine the associations between distance to BMS center and each outcome measure, controlling for demographic and clinical variables.

**Results:**

Of the 726 participants included in this study, 191 (26.3%) and 204 (28.1%) were < 20 and between 20-49.9 miles from a BMS center, respectively; 331 (46.6%) were >50 miles from a BMS center. Greater distance to BMS center was associated with white race/ethnicity (p< 0.001) and employment at time of injury (p=0.001). Greater distance to BMS center was also associated with flame injury (p< 0.001) and larger burn size (p< 0.001). There were no significant differences in length of stay or number of operations between groups. Regression analyses did not identify significant associations between distance to BMS center and VR-12 PCS, VR-12 MCS, SWL, employment at 12 months, or days to return to work.

**Conclusions:**

After burn injury, patient-reported outcome measures of physical and psychosocial function, as well as employment, do not differ based on distance to BMS center.

